# Uterine Sensitization-Associated Gene-1 in the Progression of Kidney Diseases

**DOI:** 10.1155/2021/9752139

**Published:** 2021-08-07

**Authors:** Xiaohu Li, Wenlong Yue, Guiwen Feng, Jinfeng Li

**Affiliations:** Department of Kidney Transplantation, The First Affiliated Hospital of Zhengzhou University, Zhengzhou 450052, China

## Abstract

Uterine sensitization-associated gene-1 (USAG-1), originally identified as a secretory protein preferentially expressed in the sensitized rat endometrium, has been determined to modulate bone morphogenetic protein (BMP) and Wnt expression to play important roles in kidney disease. USAG-1 affects the progression of acute and chronic kidney damage and the recovery of allograft kidney function by regulating the BMP and Wnt signaling pathways. Moreover, USAG-1 has been found to be involved in the process of T cell immune response, and its ability to inhibit germinal center activity and reduce humoral immunity is of great significance for the treatment of autoimmune nephropathy and antibody-mediated rejection (AMR) after renal transplantation. This article summarizes the many advances made regarding the roles of USAG-1 in the progression of kidney disease and outlines potential treatments.

## 1. Introduction

Acute kidney injury (AKI) is a clinical syndrome involving acute renal dysfunction caused by ischemia-reperfusion, drug toxicity, and sepsis [1]. If AKI is not treated in a timely manner, it may further develop into chronic kidney disease (CKD) or even end-stage renal disease (ESRD), during which renal tubular epithelial cells (TECs) and the endothelium are damaged more severely, leading to renal tubular damage with severe interstitial fibrosis [2]. Unfortunately, there is currently no effective treatment for reversing ESRD [3]. Kidney transplantation has gradually become the best treatment option for patients with ESRD. However, the survival of the transplanted kidney is restricted by antibody-mediated rejection (AMR) after kidney transplantation. Tubule atrophy and interstitial fibrosis are also risk factors for progressive graft dysfunction [4]. Uterine sensitization-associated gene-1 (USAG-1) is a newly discovered important cell signaling regulator that has been reported to play a key role in kidney injury, tooth development, hair growth, limb morphology, and trigeminal ganglion formation [5–8]. USAG-1, as a regulator of the bone morphogenetic protein (BMP) and Wnt signaling pathways, is abundantly expressed in the kidney and expected to repair renal tubular damage and reverse the process of interstitial fibrosis [5, 9, 10]. Furthermore, recent studies have shown that USAG-1 participates in the germinal center (GC) reaction and inhibits humoral immunity. These findings may provide new directions for the future development of treatments for AMR after kidney transplantation, as well as vaccines and therapies for autoimmune kidney diseases [11].

## 2. USAG-1 in Kidney Disease

### 2.1. Discovery and Identification of USAG-1

**Figure 1:**
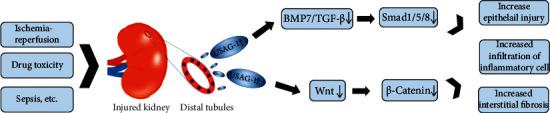
Theoretical effects of USAG-1 in renal diseases. The secretion of USAG-1 in the distal tubule increases when the kidney is damaged by ischemia reperfusion, drug toxicity, sepsis, or other factors. It aggravates tubule damage, inflammatory cell infiltration, and interstitial fibrosis by affecting the activity of the BMP7/TGF-*β* and Wnt/*β*-catenin signaling pathways.

USAG-1 (also known as WISE, sostdc1, and ectodin) is a secreted protein with a molecular weight of 28-30 kDa that contains a C-terminal cysteine knot-like domain. Laurikkala and colleagues first discovered this gene, which they named ectodin, while studying enamel junctions [12]. Simmons and Kennedy identified USAG-1 as a novel gene expressed in the endometrium of rats during maximum sensitization/uterine receptivity [13]. In addition, USAG-1 has been reported to be downregulated in renal tumors as a tumor suppressor gene, while it is highly expressed in normal kidneys [14]. Some studies have shown that USAG-1 is abundant in renal tubules and teeth at the later stage of embryonic development. In adult tissues, USAG-1 is most highly expressed in the kidney and is mainly concentrated in distal collecting duct epithelial cells, while its expression is relatively low in other tissues and organs (Figure 1) [15–17]. Yanagita et al. found that USAG-1 is a novel BMP antagonist that is highly expressed in the kidney and acts synergistically with BMP7 in developing and adult kidneys [18]. In addition, USAG-1 has been reported to act as a Wnt regulator, modulating the balance of Wnt signaling through Wnt coreceptor complex integration inputs [19].

### 2.2. USAG-1: A Novel BMP7 Antagonist Expressed in the Kidney, Accelerates Tubular Injury

BMP is a developmentally conserved signaling molecule that has been proven to belong to the transforming growth factor-*β*-1 (TGF-*β*) superfamily [1]. It was originally named for its ability to induce the formation of bone, cartilage, and multiple ectopic bones. It was found to play an important regulatory role in proliferation, differentiation, apoptosis, embryonic development, and organ formation in most cells [20]. Bone morphogenetic protein 7 (BMP7) is a 35 kDa homodimeric protein, also known as osteogenic protein-1 (OP-1) [21, 22]. Dudley et al. found that kidney development is severely delayed in BMP7-deficient mice and that these mice generally die within a short period of time after birth [23]. Subsequently, in several other animal models, such as models of acute ischemic injury, diabetic nephropathy, and chronic kidney injury, BMP7 expression was shown to be downregulated and then to gradually increase with further development of disease [24–26]. Thus, BMP7 may play an indispensable role in the normal development of the kidney. Multiple reports have demonstrated that BMP7 alleviates acute and chronic kidney injury, including by reducing apoptosis and necrosis of renal TECs, inhibiting the expression of inflammatory cytokines, reducing inflammatory cell infiltration, and reversing the progression of renal fibrosis [1, 24, 27]. Unfortunately, due to the widespread distribution of BMP receptors, exogenous administration of BMP may cause additional damage in other tissues. However, the activity of BMP has been found to be regulated by BMP antagonists, which have targeted effects by directly binding to BMP and inhibiting its binding to the corresponding receptor. Thus, USAG-1 may be a new target for the treatment of kidney disease [28].

Yanagita et al. compared the expression of USAG-1 and other BMP antagonists in the adult kidney by modified real-time PCR and in situ hybridization and found that USAG-1 is the most abundant BMP antagonist in the adult kidney [29]. Recombinant USAG-1 protein can directly bind to BMP and inhibit BMP-mediated alkaline phosphatase (ALP) activity in C2C12 and MC3T3-E1 cells in a dose-dependent manner [28]. USAG-1 knockout mouse models of AKI caused by cisplatin and chronic kidney injury caused by unilateral ureteral obstruction (UUO) were found to have significantly longer survival times and more complete renal function preservation than wild-type mouse models of these conditions. Moreover, the application of an anti-BMP7 neutralizing antibody can eliminate the renal protective effect of USAG-1 deficiency, suggesting that USAG-1 can block the repair of renal injury by antagonizing BMP7 [29]. Subsequent reports have proven that USAG-1 always colocalizes with BMP7 in the developing glomerulus and that its expression decreases during renal tubular injury and increases during renal tubular regeneration [5]. In addition to being a possible therapeutic target for kidney disease, USAG-1 may also be used as a biomarker of renal prognosis—the expression of USAG-1 in a renal biopsy specimen from a mouse model of CKD is related to the prognosis of renal function [5, 28]. Tanaka et al. found that USAG-1 may enhance the expression of MMP-12 in the glomerulus by inhibiting the inhibitory effect of BMP7 and aggravate the progression of glomerular disease in Alport syndrome, while the genetic ablation effect of USAG-1 greatly reduces disease progression in Col4a3–/– mice (a model of human Alport syndrome) and preserves kidney function [30]. Xia et al. found that febuxostat alleviates renal dysfunction and tubulointerstitial fibrosis in rats with UUO by inhibiting USAG-1 expression and activating the BMP7-SMAD1/5/8 pathway [31, 32]. In subsequent studies, it was demonstrated that USAG-1 expression is downregulated in vitro in a Madin-Darby canine kidney (MDCK) cell model, which helped reverse TGF-*β*1-induced epithelial-mesenchymal transformation (EMT) [9]. Smad1/5/8 is a key intracellular protein for transducing BMP7 signals, and phosphorylation of Smad1/5/8 is also the central downstream event in the BMP signal transduction pathway [5, 9, 33]. As a specific antagonist of BMP7, USAG-1 is also an antagonist of TGF-*β* family ligands, which are involved in promoting EMT by at least partially inhibiting the activity of the Smad1/5/8 signaling pathway (Figure 1) [9, 34]. This finding is consistent with the working model proposed by Yanagita et al., in which USAG-1 and BMP7 play a role in EMT [29]. Interestingly, similar to the perspective above, the loss of USAG-1 promotes the expansion and differentiation of mesenchymal cells (MSCs) during fracture repair, thereby accelerating the healing of fractures [35].

### 2.3. USAG-1: Activator and Inhibitor of Wnt Signaling

The Wnt signaling pathway is a developmental signaling pathway that can promote embryonic development of the kidney, repair kidney damage, and regulate the formation of various structures in the kidney [36–38]. USAG-1 has been reported to activate and inhibit Wnt signaling in a context-dependent manner. USAG-1 not only interacts with the Wnt coreceptor lipoprotein receptor-related protein 6 (LRP6) but also competes with Wnt8 by binding with LRP6 [19]. According to Qian et al., USAG-1 inhibits endogenous Wnt-induced *β*-catenin-dependent transcriptional activity in a dose-dependent fashion and directly affects the expression of E-cadherin and *α*-smooth muscle actin (*α*-SMA) in renal epithelial cells and mesenchymal fibroblasts [39]. USAG-1 can enhance Wnt3A signal transduction and inhibit Wnt1 and Wnt10b to a certain extent in in vitro cell experiments, which is consistent with previously published data showing that USAG-1 activity depends on the type of Wnt [6, 19, 39].

Chronic allograft injury (CAD) is a disease characterized by renal tubule atrophy, interstitial fibrosis, and glomerular lesions, and it seriously affects the survival of renal transplant recipients [40]. Wnt signaling is known to regulate various morphogenetic pathways, such as cell migration, cell proliferation, and cell fate determination, and the dysregulation of these pathways is closely related to the occurrence of CAD. Therefore, increasing normal Wnt signaling and minimizing abnormal Wnt signaling may be potential strategies for therapeutic interventions in CAD and other progressive kidney diseases [41–43]. Seifert et al. found that the Wnt signaling pathway was associated with microvascular injury and renal allograft failure in a residual clinical biopsy conducted 10 years ago, and further mechanistic studies may identify the Wnt signaling pathway as a new target for the diagnosis and treatment of CAD [44]. In a rat kidney transplantation model, allogeneic transplanted kidneys were found to have Wnt signaling components, and the administration of an anti-USAG-1 antibody can significantly improve the recipient's transplanted kidney function. This may be related to the increase in total *β*-catenin expression induced by the anti-USAG-1 antibody. In addition, long-term prophylactic treatment with a rat anti-USAG-1 antibody can reduce CD68^+^ macrophage and CD8^+^ T cell infiltration, alleviate renal tubular damage and interstitial fibrosis, and reduce the degradation of graft structure [39]. Briefly, USAG-1 expressed in the kidney can cause renal allograft dysfunction by promoting renal tubular atrophy and interstitial fibrosis. Anti-USAG-1 antibodies have certain clinical significance for the treatment of CAD. However, the specific mechanism is still unclear, although we speculate that it may be related to regulation of the Wnt signaling pathway by USAG-1 (Figure 1) [39, 44].

### 2.4. USAG-1 and T Cell Immune Response

Loss of immune tolerance to autoantigen typifies most autoimmune kidney diseases, and the production of autoantibodies and infiltration of peripheral immune cells are typical pathological features of these autoimmune nephropathies. As crucial drivers of autoimmunity and associated organ injury, T cells play a central role in the regulation of immune responses. An enhanced understanding of the biochemistry and molecular biology of T cells in patients with autoimmune kidney disease will provide a unique opportunity for the identification of therapeutic targets for autoimmune kidney disease. Interestingly, USAG-1 has been found to be closely associated with the T cell immune response in recent studies.

In addition to its being involved in the progression of acute chronic kidney injury, USAG-1 is also associated with the development of several diseases, such as colorectal cancer, non-small-cell lung cancer, thyroid cancer, breast cancer, and gastric cancer [45–49]. In view of the important influence of USAG-1 on organ development and tumor formation, some researchers have speculated that USAG-1 may be involved in the T cell immune response. T follicle helper (TFH) cells are CD4^+^ T cells that are well known for their ability to assist in the production of B cell antibodies in the GC of lymphatic organs and enhance the B cell memory response [50]. USAG-1 has been reported to be expressed in both TFH cells and reticular fibroblast subsets [51]. The expression of USAG-1 in TFH cells is upregulated 7 days after immunization with sheep red blood cells (SRBCs) [52]. It was demonstrated in a mouse model of acute lymphocytic choroidal meningitis virus (LCMV) infection that USAG-1 is selectively expressed in TFH cells, but the authors found that the presence of USAG-1 is not essential for the differentiation or effector function of TFH cells during acute viral infection [15]. A recent report identified a distinct subpopulation of TFH cells characterized by USAG-1 expression. USAG-1-producing TFH cells can promote the differentiation and maturation of T follicular regulatory (TFR) cells [11]. In contrast to TFH cells, which trigger the GC response, TFR cells, which are newly discovered regulatory T cells (Tregs) that express Foxp3, can inhibit the GC response and humoral immunity [53]. Therefore, unlike USAG-1^−^ TFH cells, USAG-1^+^ TFH cells cannot help B cells produce antibodies. This is mainly because USAG-1 inhibits the transcriptome controlled by *β*-catenin, thereby preventing the differentiation and maturation of TFR cells [11]. In addition, the Wnt-*β*-catenin signaling pathway has been shown to be involved in the occurrence and development of a variety of autoimmune kidney diseases [54, 55]. Blocking Wnt stimulation with exogenous USAG-1 to promote commitment to the fate of TFR cells may be a new therapeutic direction for autoimmune kidney diseases.

AMR after kidney transplantation has become a major obstacle affecting the long-term survival of transplanted kidneys [56, 57]. When AMR occurs, B cells and TFH cells in lymphoid tissues are activated by the alloantigen. The persistent immunological damage caused by the production of donor-specific antibodies (DSAs) by GC B cells through mediating the secretion of IL-21 is the main cause of renal graft function deterioration and even renal graft loss [58–61]. Tregs are potent inhibitors of immune function, playing a unique role in alleviating long-term inflammation and maintaining immunological self-tolerance [62]. Classical Wnt signaling can inhibit the function of Treg cells [63], while USAG-1 can inhibit the canonical Wnt-*β*-catenin pathway [6, 19]. In consideration of the above findings and the inhibitory effect of USAG-1^+^ THF cells on the GC reaction and humoral immunity, we speculate that further mechanistic studies will identify potential approaches for the prevention and treatment of AMR in renal transplantation.

Furthermore, NK cells, as innate lymphocytes, play an important role in eliminating viral infections and cancer cells. Millan et al. found that USAG-1 expressed by T cells and stromal cells can affect NK cell maturation and cytotoxicity by regulating Wnt signaling in NK cells [16].

## 3. Summary

As a modulator of the BMP and Wnt signaling pathways, USAG-1 is involved in the development and progression of kidney disease. We summarized the theoretical effects of USAG-1 in renal disease (Figure 1). BMP7 plays a vital role in the development and regeneration of the kidney [64]. USAG-1 can inhibit the interaction of BMP and its receptor by directly binding BMP ligands, thereby limiting the activity of BMP [18]. USAG-1 is a kidney-specific gene. Compared with exogenous administration of BMP7, which may cause extrarenal side effects, therapeutic option targeting USAG-1 enables more specific targeting and minimize adverse effects [65]. Wnt signaling is closely related to the occurrence and development of natural and transplanted CKD [44]. Wnt signaling also plays an important role in regulating microangiogenesis and damage repair and reducing immune cell infiltration [66–68]. More significantly, as the role of USAG-1 in the T cell immune response has been gradually confirmed, targeted regulation of the USAG-1-Wnt signaling pathway may be a potential approach for treating autoimmune kidney disease and preventing the occurrence of AMR. Of course, the existing small animal models and cell experiments are far from sufficient, and more evidence and further mechanistic exploration are needed to provide a theoretical basis for the use of treatments targeting this protein.
